# OECD Case Studies of Integrated Regional and Strategic Impact Assessment: What Does ‘Integration’ Look Like in Practice?

**DOI:** 10.1007/s00267-022-01631-w

**Published:** 2022-04-06

**Authors:** Lauren Arnold, Rob Friberg, Kevin Hanna, Chris G. Buse

**Affiliations:** Centre for Environmental Assessment Research, University of British Columbia-Okanagan, Kelowna, BC Canada

**Keywords:** Regional environmental assessment, Strategic environmental assessment, Integrated impact assessment, Land-use planning, Environmental impact assessment

## Abstract

Increasingly, protocols for assessing the impacts of land-uses and major resource development projects focus not only on environmental impacts, but also social and human health impacts. Regional and Strategic Environmental Assessment (RSEAs) are one innovation that hold promise at better integrating these diverse land-use values into planning, assessment, and decision-making. In this contribution, a realist review methodology is utilized to identify case studies of “integrated RSEA”—those which are strategic, have a regional assessment approach, and seek to integrate environmental, community and health impacts into a singular assessment architecture. The results of a systematic literature review are described and six RSEA-like case studies are identified: Kimberly Browse LNG SEA; HS2 Appraisal of Sustainability; Lisbon International Airport SEA; Beaufort Regional Environmental Assessment; Nordstream 2 Transboundary EIA; and the Portland Harbour Sustainability Project. The case studies are examined according to their unique contexts, mechanisms and outcomes of their assessment protocols to determine the degree to which they consider more than environmental valued components, and the means by which they were included. Findings suggest that RSEA has a contentious relationship with the integration of more than environmental values, but that there are significant lessons to be learned to support project planning, especially for assessment contexts characterized by large, transboundary projects.

## Introduction

Decades of scholarship and practice has aimed to assess and understand the environmental impacts of diverse interacting land-uses and major resource development projects, and many questions remain around best practices for assessing impacts to environmental, social, and health values across time and space, and the governance and sustainability challenges associated with such imperatives (Parkes et al. 2019; Sinclair et al. 2008; Therivel 2012). Several assessment frameworks have emerged in attempts to capture complex interactions between environmental, social, and health systems. Regional and Strategic Environmental Assessment (RSEAs) are one such innovation that hold promise at better integrating diverse land-use values into planning, assessment, and decision-making. RSEAs are an important pilar of Canada’s new *Impact Assessment Agency of Canada* (2019), which provides new guidance on how to trigger an RSEA, and significantly expands the purview of impact assessors and proponents to include a variety of land-use values across environmental, community (e.g. cultural, socioeconomic) and health domains.

In this paper, we present findings from a ‘realist review’ of literature, with a specific focus on six case examples of ‘integrated’ RSEAs. The term ‘integrated’ is meant to signal a specific focus on assessments that are inclusive of environmental, community and health values (Gillingham et al. 2016). We explore RSEA-like assessments drawing from OECD countries to build practical knowledge about implementing integrated RSEAs. The objectives are to [1] identify how ‘integration’ is understood in the nascent literature, [2] examine examples of successfully implemented RSEA-like case studies that incorporate regional perspectives and strategic orientations and take an integrated land-use value approach in their assessment (e.g. environmental, community and health values), and [3] to outline opportunities and challenges to support effective integrated RSEA in the future.

We briefly introduce the state of the literature on RSEA before presenting our review methodology. We then provide the results of our article and case study analysis focused on the context (assessment rationale, framing, and scope), mechanism (approach and frameworks for the assessment and values integration), and the outcomes of integrated assessments. The discussion section outlines the key findings, recommendations, and lessons learned to support future implementation of integrative RSEAs, with implications for project-specific environmental impact assessment (EIA).

### What is RSEA?

The notion of an RSEA emerged in the Canadian context as an attempt to merge regional assessment techniques with strategic goals that seek to not only understand project-specific impacts, but the broader implications of a project or multiple and overlapping land-uses (Gunn and Noble 2009). It can be thought of as a complement to project-based EA, or as stand-alone processes for evaluating the impacts of a proposed initiative, policy or program. The ‘regional’ component speaks to a requirement to account for a broader geographic area than any one project’s footprint, while also considering the influence of past, present and future developments in an assessment area (Sadler 2011; Therivel 2012). In order for an assessment to be ‘strategic’, it must be proactive in considering alternative development pathways in the context of a broad vision for a project, or collection of projects, that can be evaluated through goals and objectives (Noble 2000; Noble and Nwanekezie 2017; Stoeglehner 2019; Unalan and Cowell 2019). The notion of an RSEA has seen limited uptake outside of the Canadian context, and confusion arises because other assessment iterations may emulate similar characteristics. For instance, Strategic Environmental Assessments (SEA) can be regional in scope bounded by a certain geographic boundary, and regional EIAs or Cumulative Effects Assessments (CEA) can be strategic in their application.

Impact assessment disciplines are increasingly expected to be “integrated” and to consider impacts and interactions across environmental, social, and health systems. Though there are some critiques of this imperative and the effectiveness of assessment frameworks that have been developed to implement it (Morrison-Saunders and Fischer 2006), there is a growing body of work exploring these frameworks and the need for integration of diverse land use values and social systems (Bond et al. 2012; White and Noble 2013), and recent assessment regulations in Canada have emphasized integration and a focus on social and environmental sustainability (*Impact Assessment Act*, 2019; see Impact Assessment Agency of Canada: “Practitioners Guide”). Through its earliest iterations to the present, RSEAs have made attempts to leverage insights from environmental, social and health impact assessment methods (Gunn and Noble 2009). Like project-based EIA, RSEAs typically depend on the assessment of valued components, or the indicators that benchmark or monitor environmental, social and health conditions as related to project-specific impacts, though RSEAs provide unique opportunities to integrate numerous land use values into a single assessment architecture and assess cumulative effects and their implications for a range of policy objectives across space and time (Bidstrup et al. 2016; Bidstrup and Hanson 2014; Harriman and Noble 2008).

The state of knowledge surrounding regional and strategic assessment frameworks is growing and provide a helpful literature base for supporting the development and implementation of RSEAs. Reviews of current methods and guidance for implementation exist speaking to both regional and strategic goals (Fischer and Onyango 2012; Gunn and Noble 2009; Noble et al. 2012; Tetlow and Hanusch 2012; Therivel 2012; White and Noble 2013; Zhang et al. 2013); and jurisdictional reviews of policies supporting both regional and strategic environmental assessment have been completed at the national and international level (Chaker et al. 2006; Fischer and Onyango 2012; Harriman and Noble 2008; Lobos and Partidario 2014). However, to date, there have been few evaluations of RSEA protocols in use that systematically account for the contexts in which they are implemented, and little exploration into the effectiveness of specific methods and/or best practices for analyzing interrelated environment, social and health impacts over time. Accordingly, existing protocols may not be reflective of contemporary global priorities such as climate change, current debates over criteria for social license to operate, and/or Indigenous rights and title. While there is a great deal of research arguing that integrated impact assessment is needed, and evidence that RSEA has potential to redress this, there is limited practical guidance on how to carry out such assessment (Noble et al. 2019).

## Methods

To understand the elements that can drive successful integrated RSEA implementation, we used a realist review strategy. Realist reviews are a form of knowledge synthesis that analyze the context(s), mechanism(s) and outcome(s) for social and public policy issues characterized by a high degree of complexity (Pawson 2006). A realist review adds an explanatory focus to a systematic review (Pawson and Bellamy 2006) by building an understanding what works, to what degree, for whom, and in what circumstances (Greenhalgh et al. 2011; Pawson 2006). The ‘realist’ aspect of the ‘realist review’ is rooted in a social science paradigm that recognizes objective realities are mediated by human perspective (Olsen 2010). Realist reviews therefore embrace methodological pluralism—combining both quantitative and qualitative reporting—and support comparative research by analyzing the contextual features of case examples that may be of relevance to context(s) of interest (Edgley et al. 2016). To that end, a realist reviews may articulate the underlying assumptions about how an RSEA is meant to work, what its intended outcomes are, and empirical analysis of evidence that supports, contradicts or modifies these assumptions.

**Fig. 1:**
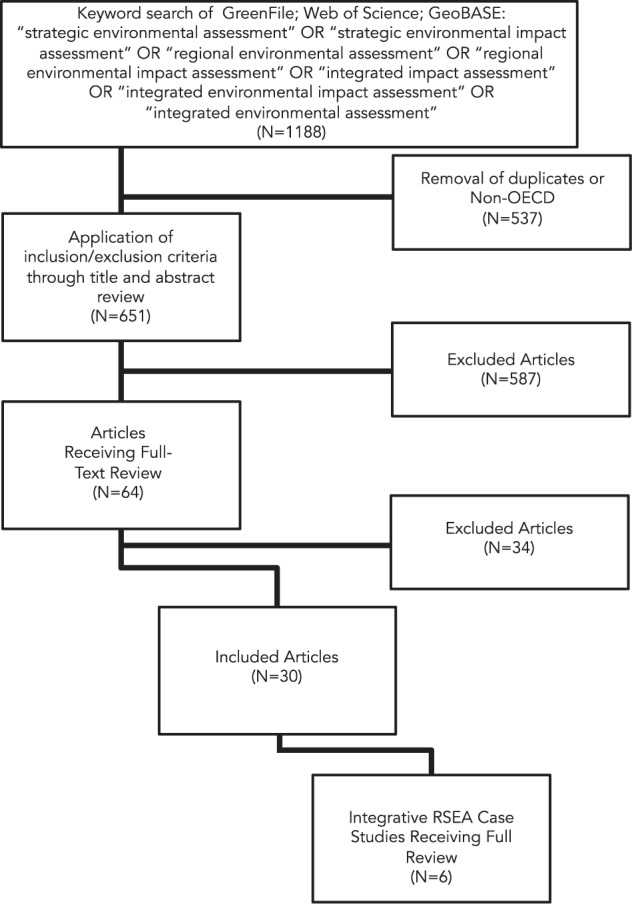
Case study selection process

Our review had two phases. In phase 1 we conducted a review of the scholarly literature from the past 10 years that report on integration (i.e. bridging environmental, socioeconomic and health impacts) within established or on-going RSEA-like assessments in OECD countries. Using a systematic search process of three scholarly databases (see Fig. 1), we then identified 30 articles as in scope and contributing to the objectives of the review. This corpus (*N* = 30) served as the body of literature to determine how ‘integration’ is understood in RSEA-like literatures. We included only articles in English, published between 2010 and 2019. We excluded articles that were strictly theoretical in their orientation and not linked to practical assessment. We found that much scholarly literature has been dedicated towards the conceptualization of RSEAs, with limited practice-based evaluations of actual studies. Indeed, our study surfaced 328 references that were conceptual contributions, rather than practice-based contributions to the RSEA discourse. The 30 in scope articles served as the basis by which to understand how ‘integration’ of diverse values is considered in the context of RSEA-like assessments. Full text reviews were completed by the research term using an annotation template that included descriptive information for each article, and reflections on the principle contribution, the context(s), mechanism(s), and outcome(s) of each study, and general observations on how integration was approached.

Phase 2 examined practice-based documents (e.g. assessment reports, policy briefs, grey literature studies) of RSEA-like case studies to extract lessons for achieving integration of land-use values in RSEA. Case studies were identified during the full text reviews of articles during Phase 1 and by reviewing the reference lists of key articles pointing to RSEA-like examples. This process identified six RSEA-like case studies. Importantly, these case studies are not themselves RSEAs in their regulatory orientations, but were selected as emulating regional and strategic principles and based on their level of values integration and consensus of the research team.

Case study reviews were divided between two researchers. An annotation template was completed for each case study which included an annotated bibliography of relevant documents, assessment reports, and published articles associated with the case, and a number prompting questions for reflections on its context, mechanisms, outcomes, and approach to integration (see Box 1). An excel spreadsheet was created for each case study in order to track the Valued Components (VCs) included and the indicators used to evaluate them, where applicable. Once the review templates were filled out the results and any challenges in answering review questions were discussed with the full research team.

Box 1: Case study analysis framework **How is the context of the assessment characterized?**

•What was the rationale for the assessment?

•What are the contextual features of the region being assessed (e.g. spatial scale, demographics, economic considerations, strategic considerations, etc.)

**What were the mechanisms for implementing the Regional/Strategic EA?**

•Named policy protocols or assessment protocols, data collection approaches?

•What were the suite of research methods utilized?

•What kinds of stakeholders were involved, if any, and how?

•Is there any reference to making decisions about how to weight trade-offs between valued components (what are they, how was this accomplished)?

**What, if any, are the stated outcomes of the RSEA, and how do they mesh with any stated outcomes of the project?**

•Is there any evaluative information on the successes and failures of the RSEA?

•Any future practical guidance that speaks to the challenges/opportunities faced in this case of RSEA implementation?

## Results

### What Does ‘Integration’ Look Like in the RSEA-Like Literature?

Our Phase 1 review surfaced 30 resources that spoke to examples of ‘integration’ in environmental assessments that encompass either a strategic or regional component. Of these, 12 were case study articles of RSEA-like assessments exhibited integration of multiple values, and 18 were articles describing methodological innovations to achieve integration. Integration within this body of literature has several meanings and interpretations. Our included sample of integrated RSEA-like case studies demonstrated a greater degree of values integration than the sample of methodologically innovative studies, according to our definition of integration as referring to incorporation of multiple community, health, and environmental values.

Among the of articles, 13 integrated both environment and social values, 2 integrated environment and health values and no articles focused solely on social and health values (Table 1). Only 4 articles focused on merging environmental, social and health values into a singular impact assessment architecture, though even in these cases where diverse values integration occurred, environmental components maintained a central focus in assessment, with comparably fewer social and health values included. Of those articles that had a principally methodological focus (*N* = 18) there were 10 articles that focused solely on the evaluation of multiple environmental values and one that focused solely on multiple social values. In these articles the term integration was used in reference employing one of more research methods, or merging two or more environmental values, which demonstrates the multiple interpretations of integration within the literature base.

**Table 1:** Values integration observed during phase 1

Domains of values integration					
	Total articles	Environment + Social	Environmental + Health	Social + Health	Environment + Social + Health
Integrated RSEA-like case studies	12	8	2	0	2
Methodological Innovation	18^a^	5	0	0	2

^a^Of those articles focused on methodological innovation, 10 described integration as the incorporation of multiple environmental values, and 1 described it as the incorporation of multiple social values

Across the set of articles we found that the interpretation of integration remained narrow, and as a whole we found a lack of detailed descriptions as to the context, mechanisms, and outcomes of integrated RSEA implementation. In the second phase of these review we explored selected case studies and their source material in more detail with the aim of uncovering more detailed descriptions.

### Description of Included Case Studies

This section provides an overview of each of the 6 selected case examples of integrated RSEA (see Table 2). Collectively, the case studies span several countries and industries. Selected case studies were not necessarily carried out as formal RSEAs. We consider our selected case studies as “RSEA-like” processes, following Dalal-Clayton and Sadler (2017) and Noble (2009)’s descriptions of “SEA type” assessments, in that they are examples that incorporate strategic orientations, regional scope, and applications of RSEA principles and methods, but may not be formally defined as RSEAs. Some of our case studies were conducted under formal regulatory frameworks as SEAs or other assessments, and some were completed independent of specific legislative requirements. All case studies included a strategic orientation and goal of contributing to some level of planning decisions. Some were initiated with an explicit ecological site, national, or multi-national focus and others involve evaluation of multiple defined sites for a specific action.

**Table 2:** Selected case studies

Case study	Country	Year complete	Assessment focus	Spatial scope
Kimberly Browse LNG SEA	Australia	2010	Impact assessment and site selection for a potential LNG precinct	West Kimberly coast—regional area and multi-site analysis
HS2 Appraisal of Sustainability	United Kingdom	2011	Evaluating alternatives and impacts of a potential highspeed railway line and infrastructure	Multi-city. Proposed 225 km of railway and infrastructure
Lisbon International Airport SEA	Portugal	2008	Evaluating alternatives and site selection for expanding airport and runway capacity	Municipal and Regional. Airport model selection, site selection, and comparative analysis of current and selected airport site
Beaufort Regional Environmental Assessment	Canada	2016	Assessment of regional and cumulative effects of the offshore oil and gas industry	Beaufort Sea
Nordstream 2 Transboundary EIA	Transboundary	2017	Assessing of transboundary impacts of undersea natural gas pipeline	Multi-country. 1200 km of pipeline and offshore/onshore facilities.
Portland Harbour Sustainability Project	United States	2018	Evaluation of remediation options for Portland Harbour Superfund site	Municipal. Defined remediation site with explicit focus on regional economic development opportunities.

#### Kimberly Browse SEA

The Kimberly Browse SEA was initiated to assess a proposed Liquified Natural Gas (LNG) precinct on the Kimberly coast of Western Australia. This single common user precinct was intended to prevent piecemeal development by individual companies and to minimize potential cumulative effects. The scope of this SEA was limited to the LNG industry and was completed under the national *Environmental Protection and Biodiversity Conservation Act* (1999). The precinct would consist of processing facilities and infrastructure with a capacity of up to 50 million tonnes per annum (Western Australian Government and Australian Government 2010). A key objective of this assessment process was site selection (Western Australian Government and Australian Government 2008). Over 40 potential sites were considered for the precinct before selecting the James Price Point location (Western Australian Government and Australian Government 2008). The location for the proposed precinct is entirely on unallocated crown land with adjacent lands and waters that are subject to a registered claim under the Commonwealth Native Title Act (Western Australian Government and Australian Government 2008). The SEA’s conclusions emphasized the benefits of the precinct and its role in avoiding potential negative impacts by preventing ad hoc development by multiple proponents (Western Australian Government and Australian Government 2010). In addition, the site selection process was credited with helping to avoid many of the potential adverse impacts. However, there has been ongoing controversy around the environmental review and the extent to which the assessment and the site selection process included meaningful engagement with the public and Indigenous communities and considered potential Native title (Mills 2019; O’Faircheallaigh 2009; 2010; 2011) and the precinct has yet to be constructed.

#### HS2 Limited Appraisal of Sustainability

In 2011 HS2 Limited completed an Appraisal of Sustainability for a proposed highspeed rail connection between London and the West Midlands (HS2 Limited 2011; 2012). This development includes 225 km of new railway, and associated station and interchange infrastructure (HS2 Limited 2011). An Appraisal of Sustainability in the United Kingdom (UK) is a process parallel to SEA and was developed to integrate requirements of the European Union SEA directive (2007) (Carvahlo et al. 2017). It is specifically applied during the planning phase with a key goal to evaluate reasonable alternatives to a project and outline potential impacts prior to the completion of an EIA (HS2 Limited 2011). The Appraisal of Sustainability was oriented around the extent to which the project would support and/or conflict with objectives for sustainable development which had been previously outlined in the United Kingdom Sustainable Development Strategy (2005). The Appraisal was completed in 2011 and an EIA for the project was completed in 2013 receiving approval in 2017. Assessment and appraisal processes for a second for phase of this rail system are now underway.

#### Lisbon Airport SEA

Economic and population growth prompted a need to expand runway capacity at the Lisbon Airport, and surrounding urban expansion prevented this growth at the original airport location. The SEA drew on methodology and guidance developed by Partidário (2007) and was conducted under the European Union SEA Directive. The scope involved a technical feasibility and comparative assessment of potential airport models and locations (National Laboratory of Civil Engineering 2008). Academic literature published in English prompted us to examine this case study (Partidário 2007; Partidário and Coutinho 2011), but the source material for the assessment is primarily in Portuguese. A co-author who had fluency was able to review this material. The SEA was based on a “strategic sustainability approach” which included determining “critical decision factors” under which a series of indicators were identified (Partidário and Coutinho 2011). The SEA resulted in the national government reversing a previous decision about site location. This decision was later reversed, in favor of upgrades to the existing airport and conversion of a military airbase at Montijo. The final airport location and decision but the airport location decision remains controversial.

#### Beaufort Regional Environmental Assessment

The Beaufort Regional Environmental Assessment was defined as a “geographically based research program” (Beaufort Regional EA 2016). The process was centered around a goal of data generation and filling knowledge gaps related to the impacts and cumulative effects of offshore oil and gas development in the Canadian Beaufort Sea. The assessment process was launched in 2010 and a final report was published in 2016. The key focus of the assessment was to generate information to support future project-level EAs in the region and facilitate other planning processes (Beaufort Regional EA 2016). Other objectives included helping communities and stakeholders prepare for future development, strengthening government, industry, and research partnerships, strengthen engagement, and to determine economic and environmental goals (2016). The process facilitated a range of scientific studies and research under the leadership of several multi-stakeholder working groups.

#### Nordstream 2 Transboundary EIA

The Nordstream 2 project would involve twinning an existing pipeline (operational in 2012) that runs from the Baltic Coast in Russia, through the Baltic Sea, to Greifwald in Germany (Nordstream 2 2017). This would include 1200 km of pipeline with a capacity of 55 billion cubic meters of natural gas per year and the construction of offshore and onshore facilities. Under the United Nations Convention on Environmental Impact Assessment in a Transboundary Context (Espoo Convention), projects that cross a national border trigger a need to assess transboundary impacts and evaluate the project as a whole since impacts that may arise in one country can affect another (Nordstream 2 2017). This was an international scale assessment and included Parties of Origin, which are those countries where the pipeline will be located (Russia, Finland, Sweden, Denmark, and Germany) and Affected Parties which are countries that might be affected even though the project is not within their boundaries (Estonia, Latvia, Lithuania, and Poland). This assessment was undertaken alongside individual EIA processes in origin countries and was focused on providing an overarching assessment and understanding of the impacts and interactions between jurisdictions, and also generating information which could be useful for EIA processes (Eser et al. 2019; Goldthau 2016; Nordstream 2 2017). The transboundary EIA process was completed in 2017 and individual affected country EIA processes and approvals are ongoing.

#### Portland Harbour Sustainability Project

The Portland Harbour Sustainability Project concerns the Portland Harbour Superfund Site which is a 10-mile portion of the Lower Willamette River in Portland, Oregon. The Portland Harbour Superfund Site has been designated as a “mega sediment site” (remediation costs anticipated > $50 million USD) under the United States *Comprehensive Environmental Response, Compensation, and Liability Act* (1986). Environmental concerns include hazardous chemical contamination and risks to aquatic species habitats and human health (Fitzpatrick et al. 2018). The United States Environmental Protection Agency (2016) completed a “Feasibility Study” of remediation alternatives in 2016. The Portland Harbour Sustainability Project was initiated by a research team citing concerns that the Feasibility Study was did not consider sustainability from an integrated perspective that incorporated environment, social, and economic values (Fitzpatrick et al. 2018). The Sustainability Project involved the publication of four companion papers: a linked Environmental Impact and Benefit Analysis (McNally et al. 2018); A Regional Economic Impact Assessment (Harrison et al. 2018); a Values Linked Sustainability Assessment (Apitz et al. 2018); a Probabilistic Risk Assessment (Ruffle et al. 2018).

### The Contexts, Mechanisms and Outcomes of ‘Integrated’ RSEAs

Having briefly introduced the six case examples, we now describe the results of applying the realist review methodology to the case studies. The results are presented in three sections discussing case study contexts, mechanisms, and outcomes. Key observations under each section are reported in Table 3 and were determined through consensus of the research team.

**Table 3:** Context, mechanisms, outcomes for case studies—maybe a caption explaining that whether a case met a criteria was a consensus based process

Case study	Kimberly Browse LNG SEA	HS2 Appraisal of Sustainability	Lisbon Airport SEA	Beaufort Regional EA	Nordstream 2 Transboundary EIA	Portland HarborSustainability Project
**C**ontext						
Key industry focus	Liquified natural gas	Highspeed rail	Airport construction	Oil and gas	Natural gas pipeline	Site remediation
Assessment focus/rationale						
Regional project impacts	X	X	X	X	X	X
Industry assessment/planning	X		X			
Multi-industry assessment/planning				X		
Mechanisms						
Assessment completed under legal mechanism	X	X	X	X	X	
Clear approaches or assessment protocols identified	X	X	X	X	X	X
Opportunities for stakeholder participation	X	X	X	X	X	X
Integration of multiple values						
Environment	X	X	X	X	X	X
Health	X	X	X	X	X	X
Community	X	X	X	X	X	X
Values framework						
Valued component focused	X		X	X	X	X
Oriented on sustainability goals		X	X			X
Outcomes						
Effects assessment	X	X	X	X	X	X
Generating regional baseline data	X			X	X	
Sustainability assessment		X	X			X
Site selection	X		X			
Evaluation of alternatives		X	X		X	X
Procedures for monitoring and follow-up	X		X		X	X
Articulated goal to connect to other levels of assessment	X	X	X	X	X	
Management framework developed for use in other assessments	X				X	

#### Context

As per the descriptions above, case studies differed considerably in their orientations and intents. While two of the case studies were carried out as SEAs (Kimberly Browse SEA and Lisbon Airport SEA), these two examples have little in common in terms of their objectives and context apart from their SEA label. The remaining case studies represent a diverse range of assessment types: Appraisal of Sustainability, a Regional Environmental Assessment, and a unique transboundary EIA. While all 6 case studies differ in their context and orientation, each was grounded in strategic goals. For some of the cases (Kimberly, HS2, Lisbon, Nordstream 2, and Portland), this meant a process focused on site selection or evaluation of alternatives prior to a project level assessment, and for others this meant broad level assessment or information generation in anticipation of future projects and planning processes.

An important note is that 4 of the 6 (Kimberly, Nordstream 2, HS2, Lisbon) were triggered by a project or specific undertaking where the impacts were likely to be regional in nature or relevant in the context of broader sustainability objectives (Table 3). These assessments meet our criteria for an integrated RSEA in that they were intended to facilitate some level of strategic decision-making, but they remain reactionary in their framing to assess potential impacts of a defined action. In contrast, 2 of the RSEA case studies were initiated independently of any project assessment or specific endeavor. The Kimberly Browse SEA was completed to plan for an LNG precinct likely involving several distinct future projects. This assessment was however limited in scope to the LNG industry. The Beaufort Regional Environmental Assessment was also initiated independent any one project. Rather, it was prompted due to concerns about already significant cumulative impacts from the oil and gas industries in the region, and therefore adopted a regional and multi-industry focus (Beaufort Regional Environmental Assessment Final Report 2016). However, the primary objective of the assessment was to generate data and information, and there was much less emphasis on strategic decision-making than was present in other SEA and sustainability-oriented cases studies.

#### Mechanisms

We identified three ‘mechanisms’ that fuel RSEA implementation. These include: clear assessment protocols, guidance and articulated objectives; stakeholder participation; and integration of values or frameworks guiding the assessment. Each ‘mechanism’ is briefly articulated below.

##### Assessment protocols, guidance and articulated objectives

Five case studies were grounded in a regulatory assessment, though all case studies were conducted based on clear assessment protocols, guidance, and objectives. A key nuance exists in the phase of development and decision-making in which the assessments were applied. Four case studies were explicitly intended to result in strategic decision-making in the planning phase of a specific project or undertaking. For example, Kimberly Browse SEA included objectives for site selection for the precinct and guidance for future EAs (Western Australian Government and Australian Government 2010). The HS2 Appraisal of Sustainability was completed in the planning phase to evaluate alternatives and adjust the project plan in relation to sustainability objectives (HS2 Limited 2011). Lisbon Airport SEA was also specifically oriented towards site selection decision and evaluation of alternatives (National Laboratory of Civil Engineering 2008; Partidário and Coutinho 2011). These mechanisms to initiate an assessment in the planning phase and the presence of defined objectives and decisions that directly connect to future assessments and projects were important for the effectiveness of the assessment as a strategic process and decision-making tool.

For cases where these connections were not explicit, the meaningful application of the assessment in decision-making and its ability to influence other assessments and planning processes was less clear. For instance, the Nordstream 2 transboundary EIA was completed at the same time as EIA processes were being carried out in affected countries (2017). Though the assessment was intended to help inform EAs, its formal relationship to these processes was vague and specific strategic decision-making objectives were not clearly outlined. The Beaufort Regional Environmental Assessment was primarily concerned with generating information about regional and cumulative effects (2016). Decision-making and management outcomes, or specific planning related to these impacts and the oil and gas industry was not part of the assessment objectives (2016). This is not to say these assessments were not strategically useful, but rather that their effective application as a strategic decision-making tool is not explicitly supported by the assessment frameworks and protocols that guided them.

##### Stakeholder participation

All case study assessments included stakeholder participation. Participation took many forms informed by the scope and nature of the assessment. In the case of the Nordstream 2 transboundary EIA, participation demanded engagement across several countries and included over 200 consultation meetings with stakeholders, research organizations, and NGOs (2017). In the Lisbon Airport SEA, stakeholder participation was focused on engagement with experts over a fairly short assessment period (Partidário and Coutinho 2011). For the Kimberly Browse SEA (2010) and the Beaufort Regional Environmental Assessment (2016) engaging with Indigenous communities and governments and considering impacts on Indigenous rights was also specifically important and legally required under Australian and Canadian law. In the Beaufort Regional Environmental Assessment a collaborative partnership framework was developed to include Inuvialuit leadership and devoted substantial resources toward an Advisory Committee and six working groups focused on cumulative effects, climate change, social, cultural, and economic indicators, oil spill preparedness, waste management, and information management with multi stakeholder and Indigenous representation (2016). This facilitated the key goal of the assessment as a research and information generation process.

The consistency in attention to participation across the case studies is promising since trust, social participation, and engagement of diverse sectors and stakeholders (including ‘experts’ and those with significant lived experience) are all lauded as important and central practices to RSEA (Noble et al. 2019). However, our analysis revealed limited evidence that participation and stakeholder engagement are effectively used as a tool to facilitate integration of multiple environmental, community, and health values. Participation was primarily used to gauge public concern and to build relationships between stakeholders, and not necessarily to support assessments of impacts to environmental, community, and health systems or to gather information about impacts and planning priorities in these domains.

It is also important to highlight that for many of the case studies, the effectiveness of participation and its influence on the assessment was a subject of controversy. In the Kimberly Browse SEA, a stakeholder reference group was established, public and Indigenous consultation and workshops were completed throughout the assessment, and a social economic assessment and Aboriginal Social Impact Assessment was completed as part of the process (2010). However, concerns about the adequacy of this public and Indigenous involvement, including the time and resources, the attention to impacts and benefits, the adequacy of the assessments of social impacts, and the importance of broader questions related to Indigenous title have been the focus of subsequent critical research (Mills 2019; O’Faircheallaigh 2009; 2010; 2011).

##### Integration and values frameworks

A key objective of this review was to explore mechanisms for integrative RSEAs or those that include a range of environmental, community, and health impacts and values. Among our case studies, none had a sole social and health focus on integration, though all integrated across environment, health domains, and a wide variety of ‘social’ values (e.g. socioeconomic, cultural, heritage, behaviors, infrastructure, etc.).

Most of the assessments (*n* = 5) used a Valued Components (VCs) framework. This is a similar model to those often applied in project EIAs where the assessment is focused on components of the environmental and social environment that are ecologically and/or socially important and which might be impacted by the proposed undertaking. While we tracked VCs (where applicable) during the review, direct comparisons across case studies of the VCs included/excluded is not particularly informative as the nature of the projects, assessments, and objectives are so diverse. However, an observation across these VC focused assessments is that the assessment of the community, environment, and health components selected remained siloed, and there were few explicit linkages between the environmental, community, and health impacts and systems. For instance, VCs were identified under broad categories such as “terrestrial environment”, “marine environment”, “community and social environment” addressed in separate chapters and volumes of the assessment and few explicit linkages were made between these VCs and values and the methods used. Our analysis also indicated that environmental components maintained a central focus in assessment, and comprehensiveness of the social and health considerations were weak by comparison. As a whole in the assessments applied under regulatory frameworks and which employed VC values frameworks, we found little to no evidence of integrated methodology or models that incorporated included environmental, community, and/or health values together.

Three of the case studies used a sustainability-oriented values framework rather than a VC based framework. In these cases, the assessment was not solely based on identified VCs and the projected impacts on them, but included attention to broad objectives for sustainability and the extent to which the project or undertaking contributed towards achieving these objectives. For example, in the HS2 Limited Appraisal of Sustainability the project was assessed in terms of how it would support and/or conflict with defined objectives for sustainable development (2011). These 4 objectives were drawn from the UK Sustainable Development Strategy: Securing the Future (2005): “(1) Reducing GHG emissions and combating climate change (2) Natural resource protection and environmental enhancement (3) Creating sustainable communities (4) Sustainable consumption and production.” Under each of the sustainability objectives a number of specific issues were assessed/considered. For example, under the objective “creating sustainable communities” one of the issues identified was “accessibility” which was further defined as maintaining/enhancing pedestrian access, access to public transit, and public transit interchange (HS2 Limited 2011 p.30). This is a different orientation than a VC framework and assessing impacts upon defined environmental, social, and health components and determining where those impacts are significant or unacceptable.

The Lisbon Airport SEA and the Portland Harbor Sustainability project incorporated elements of both a VC and sustainability goals framework. For instance, in the Portland Harbor Sustainability Project, a specific Values Linked Sustainability Assessment was completed (Apitz et al. 2018). This process was based on indicators for stakeholder values in 3 areas: environmental quality, economic viability, and social equity. Metrics were developed to link stakeholder values to measurable indicators and were scored from maximum undesirable benefit to maximum desirable benefit, and aggregated into a sustainability pillar score which was used to evaluate remediation alternatives.

We found that in these case studies where there was a sustainability assessment and orientation, the objectives-based approach allowed for a level of integration amongst health, community, and environmental components under the broad concept of sustainability that was not evident in the exclusively VC focused assessments. In addition, these examples were arguably inherently strategic in the sense that they were linked to objectives and future planning by their design and underlying framework.

#### Outcomes

The assessment outcomes reported in Table 3 are those that were clearly articulated within the assessment documentation as outcomes of the process. We do note that a challenge exists in evaluating the effectiveness of these articulated outcomes, and whether the objectives of the assessment were actually realized, particularly for cases where little critical literature was available and our analysis was constrained to case documents and reports themselves.

All case studies included an effects assessment element, whether directly related to a proposed project or undertaking, or with a regional industry or multi-industry focus. For those assessments that utilized a VC framework, it was common to employ the use of a significance framework and the development of thresholds for each VC over which an impact was significant or unacceptable. In cases with a sustainability assessment framework, strategies for evaluation varied. In the Portland Harbor Sustainability Project for instance, a range of evaluative criteria were employed including an impact benefit analysis, a regional economic assessment (REMI Policy Insight Plus Model), and linked sustainability assessment (using weighted criteria) (Fitzpatrick et al. 2018).

All case studies included some level of data generation and information collection. None of our case study assessments were strictly a desktop study. However, only 3/6 explicitly identified the generation of regional baseline data as a goal and specific outcome of the assessment. These were Beaufort Regional Environmental Assessment, the Kimberly Browse SEA, and the Nordstream 2 transboundary EIA where the assessment was completed to prepared for a specific industry, industries, or a large-scale project involving many distinct assessment processes. An important note is that the focus of this information generation was primarily on environmental or biophysical components. As previously stated, most of the indicators and VCs identified in the case studies were environmental, and far fewer were focused on community and health values.

Key strategic objective for many of the case studies were site selection and evaluating alternatives. For two of the case studies, the Lisbon Airport SEA and the Kimberly Browse SEA site selection was a core focus of the assessment. Evaluation of alternatives was an element of 4 of the case studies, but to varying degrees. The Portland Harbor Sustainability Project was specifically designed to evaluate remediation options under an integrative sustainability lens (Fitzpatrick et al. 2018). In the Nordstream 2 transboundary EIA an evaluation of alternatives was focused on evaluating scenarios with the project and in absence of the project, and a limited discussion of alternatives for meeting energy needs, but the central focus was project impact assessment (2017).

An important observation is that with the exception of the Portland Harbour Sustainability Project, all of our case studies included articulated a goal to be used in other subsequent assessments and decision-making levels, such as EIA. A core objective of the Beaufort Regional Environmental Assessment (2016), the Kimberly Browse SEA (2010), and the Nordstream 2 transboundary EIA (2017) was to generate data that would be useful for subsequent project-EIAs and improve their efficiency and effectiveness. The Lisbon Airport SEA (Partidário and Coutinho 2011) and the HS2 Limited Appraisal of Sustainability (2011; 2012) were also intended to inform a subsequent EIA process for the project. However, in most cases, detail is lacking in terms of how this connection to other levels of assessment will be established. Our analysis revealed that only 2 of these case studies produced management frameworks specifically for use in other assessments. In most cases, the extent to which the goals to influence other assessments was realized is unclear.

Some insight can be gained by turning to the available literature, which provides critiques and evaluations of assessment outcomes. For the Kimberly Browse SEA, a key outcome of the SEA process was to establish a State Management Framework, which would apply to future projects in the LNG precinct and include a social management committee, an LNG operations and coordination committee, and a management committee (Western Australian Government and Australian Government 2010, p. 11). Ideally, this framework would provide a direct and actionable outcome that links the assessment to future project level EIAs. That said, published research has highlighted gaps in the scope and evaluation of the assessment and ongoing controversy associated with the assessment in respect to Indigenous rights and engagement. There remains considerable opposition to the LNG precinct and there are many unresolved issues remaining after the SEA process was complete. In this sense, the aim of the SEA to facilitate future project assessments was perhaps not as effective since the SEA does not seem to have adequately addressed public and Indigenous concerns (Mills 2019; O’Faircheallaigh 2009; 2010).

The Nordstream 2 Transboundary EIA produced management systems for health, safety, and environmental and social management, and there is an articulated intention to influence, and to help inform the individual EIAs that would need to be completed by each of the countries in order for the project to proceed (2017). However, it is unclear the extent to which this aspiration was realized, and specifics in terms of how this might occur is left up to the EIA process in each affected country. Similarly, while the Beaufort Regional Environmental Assessment brought together knowledge from past assessments and generated data that might be useful for future assessments it is not made clear how this information could or should be used in these assessments and whether the indicators and monitoring information generated as part of the process will be continued and kept consistent. The HS2 Limited Appraisal of Sustainability report (2011) was completed in advance of a project EIA for the highspeed rail line (2013), and it was evident that many of the project’s technical details, decision-making, and specific monitoring mitigation were left to the EIA. However, in the subsequent EIA we found only two explicit references to the Appraisal of Sustainability process where it is credited with allowing the consideration of route alternatives and also providing an initial consultation opportunity (Government of the United Kingdom 2013, p.4).

## Discussion and Conclusion

Using a two phased realist review we sought to uncover how integration is understood and carried out in RSEA-like assessments. Many of our findings in respect to the challenges associated with implementing a strategically oriented assessments and the importance of attention to participation and to assessment outcomes are not new, and we also note the challenge of comparing across a diverse range of assessment types and orientations implemented in a number of different international settings. However, this analysis does provide useful insight into how integration is understood within the existing literature base, and a set of observations about how it has been carried out that are helpful to jurisdictions seeking to implement integrated RSEA-like assessments.

In general, we note that there were few applied case studies to draw from. Most articles in our original sample focused on conceptual contributions to RSEA with limited practical guidance on how to carry out an integrated assessment. An important finding from phase 1 of our review is that the term integration is used and defined inconsistently. Some authors use the term in line with the goals of this review, to incorporate multiple land-use values into a single assessment architecture, while others describe it in relation to multiple methods, multiple environmental components, multiple assessment types, or multiple spatial scales. Further, where integration does refer to the inclusion of multiple land-use values, we found that it is often not clearly described beyond the selection of VCs or indicators across environmental, social, and health domains.

Our phase 2 analysis further revealed that the successful integration of multiple values can be limited due to siloed and/or informal assessment approaches. We found that health and community values were less attended to than environmental values and found limited evidence of integrated methodology, or models and methods that sought to account for community, health, and environmental values and the relationships between them. There is an analytic requirement for community values, and health, to be part of most RSEAs and RSEA-like assessments, and to move beyond conventional definitions of health that privilege direct, biophysical risks, to encompass indirect health risks and impacts to the determinants of human health and well-being (Beckwith 2012; Diallo et al. 2018; Douglas et al. 2011). We identified few direct linkages between health, community, and environmental domains in our case study assessments, and identified that integration is not often expressly discussed beyond simply including multiple different values and indicators. Theoretical conceptualizations of integration have been comparably more ambitious. For instance in Gillingham et al integration is presented as a goal and a perspective that includes accounting for the complexity and the interactions of environmental, social, and health systems (2016). Integrated assessment require more than the selection of diverse values and should include an understanding of how these values and systems relate to each other.

Across our case study reviews we observed different assessment frameworks in practice including valued component-based impact assessment process relying on project pathways, sustainability-based process relying on defined strategic objectives, and a combination of both. These framings offer different approaches to an assessment, but also suggest different strategies for integration. Most RSEA-like examples included in this study were implemented in ways similar to project-specific EIA, despite their regional focus. This may create conditions where RSEAs fall prey to pitfalls of being overly reliant on a single project, being principally focused on VCs, and potentially missing key dimensions of impact across time and space (Noble and Nwanekezie 2017). Our results present evidence that a sustainability assessment orientation and reference to defined objectives can support a level of conceptual integration under the broad concept of sustainability that is often missing from strictly VC based assessments.

The notion of sustainability and sustainability assessments are being increasingly leveraged in jurisdictions seeking to improve integration and inclusion of social and health values. For instance, the *Canadian Impact Assessment Act* 2019, an update to the previous *Canadian Environmental Assessment Act* 2012, reframes assessment around the concept of sustainability and purports fostering sustainability as a central objective along with including environmental, social, health, and economic factors and cumulative effects within the scope of assessments. Our results provide some support to these initiatives and suggest that an orientation towards sustainability objectives is supportive of strategic decision-making that reflects land-use planning and cumulative effects. However, definitions of sustainability are often necessarily broad, particularly in the contexts of large transboundary projects that bisect multiple aquatic and terrestrial ecosystems, as well as socio-political boundaries.

This suggests a need for clarity around decision-making and the outcomes of assessment. As a whole, we observed that no matter the specific context, clear outlining what the RSEA-like assessment framework is intended to do, what the outcome and decisions will be, and where they apply is important. Where there is ambiguity in terms of the scope and objectives, RSEAs may have difficulty in achieving their desired outcomes. Currently there is methodological diversity and limited practical guidance for RSEA, and even persistent inconsistency in terminology. At minimum RSEAs must be accountable to themselves, which requires clear articulation of the goals, scope, and policy connections and responsibility for attending to the implications and outcomes of the assessment.

Considering all of the case studies, it is also clear that participation and meaningful engagement is important not only for the accuracy of the assessment and its impact evaluations, but also for its success as a strategic decision-making or planning tool and whether the outcomes were accepted by the public and stakeholder groups. We also observed that case studies were light on how participation and engagement was or could be effectively used to support integrated assessments. Participation was utilized primarily to gauge concern and to build stakeholder relationships, which is important, but there was less direct evidence of its use to collect and evaluate information as part of an assessment methodology that could support integration and an analysis of community and health values.

We also reinforce the notion that an integrated assessment requires an interdisciplinary practice and professional capacity. Effective SEA demands regulatory and institutional support and professional capacity and leadership (Bistrup and Hansen 2014; Fischer 2010). Our results signal a need for incorporating broad expertise from multiple fields and established knowledge on understanding social and health impacts and conditions. This will be required in order to develop methods, modes of analysis and evaluation tools that support truly ‘integrated’ RSEA tools and processes.
